# Feasibility of different lymph node metastases delineation approaches in [^18^F]SiTATE PET/CT imaging in NET patients

**DOI:** 10.1186/s41824-025-00273-5

**Published:** 2025-10-30

**Authors:** Sophie Carina Siegmund, Magdalena Schöll, Vera U. Wenter, Gabriel T. Sheikh, Maximilian Scheifele, Franz Josef Gildehaus, Simon Lindner, Lalith K. Shiyam Sundar, Christine Spitzweg, Christoph J. Auernhammer, Rudolf A. Werner, Mathias J. Zacherl

**Affiliations:** 1 Department of Nuclear Medicine, LMU University Hospital, LMU Munich, Marchioninistr. 15, 81377 Munich, Germany; 2 Bavarian Cancer Research Center (BZKF), Partner Site, Munich, Germany; 3 Department of Radiology, LMU University Hospital, LMU Munich, Munich, Germany; 4 Department of Internal Medicine IV, LMU University Hospital, LMU Munich, Munich, Germany; 5 Interdisciplinary Center of Neuroendocrine Tumors of the GastroEnteroPancreatic System (GEPNET-KUM, ENETS-certified Center of Excellence), LMU University Hospital, 81377 Munich, Germany; 6 The Russell H Morgan Department of Radiology and Radiological Sciences, Division of Nuclear Medicine, Johns Hopkins School of Medicine, Baltimore, USA

**Keywords:** [^18^F]SiTATE, PET, Tumor delineation, NET, Tumor volume, Delineation

## Abstract

**Background:**

Accurate tumor delineation in somatostatin receptor (SSTR) PET/CT is imperative for quantifying tumor burden, therapy monitoring, and response assessment in gastroenteropancreatic neuroendocrine tumors (GEPNETs). This study aimed to evaluate different SSTR PET-based tumor delineation approaches, based on a lymph node metastasis as reference standard, and determine the most robust method for volume assessment. Singular, non-bulky lymph node metastases with a short axis diameter ≥ 1.0 cm from [¹⁸F]SiTATE PET/CT scans were included. Different tumor delineation methods were applied: fixed SUV thresholds, isocontours relative to SUV_max_, and individual backwards thresholding. The correlation between PET-derived volumes and CT-derived volumes (reference standard) were investigated.

**Results:**

An isocontour approach using 45% of SUV_max_ demonstrated the strongest correlation with CT-derived volumes (*r* = 0.874; r² = 0.764; *p* < 0.001), outperforming fixed SUV thresholds, e.g. SUV 4.0 (*r* = 0.727; r^2^ = 0.529; *p* < 0.001). The application of a backwards threshold approach resulted in the identification of significant variability (CoV: 59.6% for fixed SUV, 31.3% for isocontour).

**Conclusion:**

The 45% isocontour-thresholding relative to lesional SUV_max_ approach constitutes a robust and clinically applicable method for SSTR PET-based tumor delineation in GEPNET patients, irrespective of changes of SSTR avidity of reference tissues (e.g. liver). Further validation is required to establish its role in total tumor volume assessment and therapy monitoring.

## Introduction

Neuroendocrine tumors (NETs) of the gastroenteropancreatic (GEP) system frequently express somatostatin receptors (SSTRs), thus rendering SSTR-targeted PET/CT a pivotal imaging modality for staging, decision making in regard of therapy selection, and response assessment (Theodoropoulou and Stalla 2013; Fortunati et al. 2022). In this context, tumor delineation approaches play a crucial role in quantifying tumor burden, assess treatment response and monitoring disease progression. A possible biomarker from PET imaging is the total tumor volume (TTV), which has garnered mounting interest for the purposes of therapy monitoring and response assessment (Hou et al. 2021; Weber et al. 2023; Gallicchio et al. 2023). A meta-analysis showed a worse prognosis for patients with high TTVs on SSTR PET (Hou et al. 2021).

There are different SSTR radioligands available, in short, ^18^F ([^18^F]SiTATE) and ^68^Ga ligands. In comparison with ^68^Ga SSTR ligands, [^18^F]SiTATE offers advantages such as higher spatial resolution, longer half-life, and improved image contrast, rendering it particularly well suited for tumor delineation (Beyer et al. 2021; Lindner et al. 2020).

Despite the high prevalence of hepatic metastases in GEP-NET patients, the focus of this analysis was on lymph node metastases (Clift et al. 2020). Liver metastases, while common, often pose challenges in accurate segmentation due to high physiological liver uptake, diffuse infiltration, and limitations in defining measurable lesions according to RECIST 1.1 criteria (Eisenhauer et al. 2009; Garcia-Carbonero et al. 2015). In contrast, lymph node metastases allow for precise delineation, reducing segmentation variability and enabling a more robust assessment of tumor burden.

This pilot study systematically evaluates different tumor delineation approaches in [^18^F]SiTATE PET/CT by using singular lymph node metastases as a reference standard to determine a quantification method for TTV in patients with GEP-NET.

## Materials and methods

### Study design and patients

This study was conducted at a tertiary cancer center and included 34 patients with GEPNET, who underwent [^18^F]SiTATE PET/CT imaging in clinical routine as part of a prospective registry study. The tracer was administered in accordance with the German Medicines Products Act §13(2b) and after written informed consent was obtained from all patients.

The study was conducted in accordance with the tenets of the Declaration of Helsinki and approved by the Institutional Ethics Committee of the Ludwig-Maximilians-Universität Munich (IRB #21–0102 and #24–0982). The collection of patient data encompassed general clinical characteristics.

The inclusion criteria were as follows: (i) patients with known or highly suspected (e.g. highly increased chromogranin A) GEPNET; (ii) [^18^F]SiTATE PET/CT; (iii) at least one singular located, non-bulky and SSTR-expressing lymph node metastases with a short axis diameter (SAD) of ≥ 1.0 cm on transaxial CT. Patients with neuroendocrine carcinoma (NEC) were excluded. No patient had histologically confirmed NEC at the time of imaging, and none was reclassified as NEC during follow-up. In general, 34 lymph node metastases of 34 patients were included.

### Radiopharmaceutical

The Sifalin-TATE (acetate salt) precursor was sourced from ABX Advanced Biochemical Compounds (Radeberg, Germany). Radiosynthesis was conducted using either a GRP^®^ synthesis module (Scintomics, Fürstenfeldbruck, Germany) or a NEPTIS^®^ DB platform (ORA/NEPTIS, Philippeville, Belgium), achieving high yields within 30–40 min. Quality control followed the standards of the European Pharmacopoeia, meeting the required criteria without exception (Beyer et al. 2021; Wängler et al. 2010; Schirrmacher et al. 2006). [^18^F]SiTATE was administered intravenously at a mean activity of 211 ± 46 MBq (5.7 ± 1.2 mCi).

### Imaging protocol

PET/CT scans were conducted at the Department of Nuclear Medicine, LMU Munich (Biograph mCT Flow scanner or Biograph 64 PET/CT; Siemens Healthineers, Erlangen, Germany). Imaging commenced 95 ± 30 min after tracer injection. To enhance image quality and ensure radiation protection, patients received furosemide pre-medication, unless contraindicated. Furthermore, contrast-enhanced diagnostic CT scans were acquired in the portal venous phase using iopromide (Ultravist-300, Bayer Healthcare, Leverkusen, Germany) at 1.5 mL/kg body weight. Image reconstruction utilized the iterative TrueX algorithm (3 iterations, 21 subsets, 3D Gaussian post-filter with 4 mm full width at half maximum), with a CT slice thickness of 0.3 cm.

### CT image analysis

Assessment criteria for lymph node metastases were non-bulky, singular located, a SAD ≥ 1.0 cm on transaxial CT and a distinct location without contact to other structures. The volume of the respective lymph node metastasis was manually delineated, slice-by-slice, creating a region of interest (ROI). The volume of interest (VOI) was calculated by merging the ROIs into a VOI, using a dedicated workstation (Hermes Medical Solutions, Stockholm, Sweden).

### PET image analysis

For PET analyses, an ellipsoid VOI was created for each lymph node. A number of approaches for volumetric delineation were applied to the respective VOI and correlated with the CT-derived reference volume. The following approaches were used:

i) Fixed standard uptake value (SUV) based thresholds: SUV 15.0; SUV 14.0; SUV 13.0; SUV 12.0; SUV 10.0; SUV 7.5; SUV 6.0; SUV 5.0; SUV 4.5; SUV 4; SUV 3.5.
ii) Isocontour thresholding relative to SUV_max_: 10.0%; 15.0%; 20.0%; 25.0%; 30.0%; 35.0%; 40.0%; 42.0%; 45.0%; 50.0% and 55.0%.

Furthermore, individual backwards thresholding was performed (Mittlmeier et al. 2021). For each singular lymph node metastasis, fixed threshold values were adjusted to achieve the same PET- and CT-derived volume. A dedicated workstation (Affinity 1.1.4. Hermes Medical Solutions. Stockholm. Sweden) was utilized for this purpose.

### Statistical analyses

Statistical analyses were conducted with GraphPad Prism (version 10.0.0) software. Following the assessment of normal distribution employing the Shapiro-Wilk test, the correlation between CT- and the PET-derived volumes using different thresholds was evaluated through the implementation of the Spearman and Pearson correlation coefficient. The coefficient of variation was analyzed as the standard measure of dispersion in the probability distribution. Data were visualized in scatter and Bland-Altman plots. The threshold for statistical significance was defined as a two-sided p-value of < 0.05.

## Results

### CT image analysis

The mean SAD of all lymph nodes included was 1.4 ± 0.4 cm. The median CT-derived volume was 2.4 ± 1.9 mL. Lymph node metastases were located next to iliac vessels (4/34; 11.8%), mediastinal (8/34; 23.5%), paraaortic or interaortocaval (15/34; 44.1%), inguinal (1/34; 2.9%), mesenterial (3/34; 8.8%) and retroclavicular (3/34; 8.8%).

### Volumetric correlation of different delineation approaches

#### Fixed SUV thresholds

The highest correlation between CT-derived volume and a fixed SUV threshold was achieved using a SUV of 4.0 (*r* = 0.727; r^2^ = 0.529; *p* < 0.001; *n* = 28 pairs). The other tested, fixed SUV thresholds resulted in lower correlations with the CT-derived reference (Table 1).

**Table 1:** Correlation analyses

Fixed SUV threshold	*r*	*r* ^2^	*p*-value	Number of XY pairs
SUV 15.0	0.416	0.173	0.014	34
SUV 14.0	0.526	0.277	0.001	34
SUV 13.0	0.533	0.284	0.001	34
SUV 12.0	0.550	0.302	0.001	34
SUV 10.0	0.616	0.379	0.000	34
SUV 7.5	0.663	0.440	< 0.001	34
SUV 6.0	0.625	0.390	< 0.001	34
SUV 5.0	0.639	0.409	< 0.001	33
SUV 4.5	0.577	0.333	0.001	31
SUV 4.0	0.727	0.529	< 0.001	28
SUV 3.5	0.714	0.510	< 0.001	26

Isocontour relative to SUV_max_	*r*	*r* ^2^	*p*-value	Number of XY pairs
10%	0.804	0.646	< 0.001	19
15%	0.711	0.506	< 0.001	27
20%	0.714	0.510	< 0.001	33
25%	0.795	0.633	< 0.001	33
30%	0.799	0.638	< 0.001	33
35%	0.841	0.707	< 0.001	33
40%	0.871	0.758	< 0.001	33
42%	0.849	0.721	< 0.001	34
45%	0.874	0.764	< 0.001	34
50%	0.859	0.738	< 0.001	34
55%	0.816	0.665	< 0.001	34

Backwards thresholding	*r*	*r* ^2^	*p*-value	Number of XY pairs
SUV 8.9	0.617	0.380	< 0.001	34
SUV 32%	0.718	0.515	< 0.001	32

#### Isocontour relative to SUV_max_

The results demonstrated the highest correlation when using a PET volume isocontour of 45% SUV_max_ (*r* = 0.874; r^2^ = 0.764; *p* < 0.001; *n* = 34 pairs). The other isocontours that were tested demonstrated lower correlation to the CT-derived volume (Table 1).

**Fig. 1:**
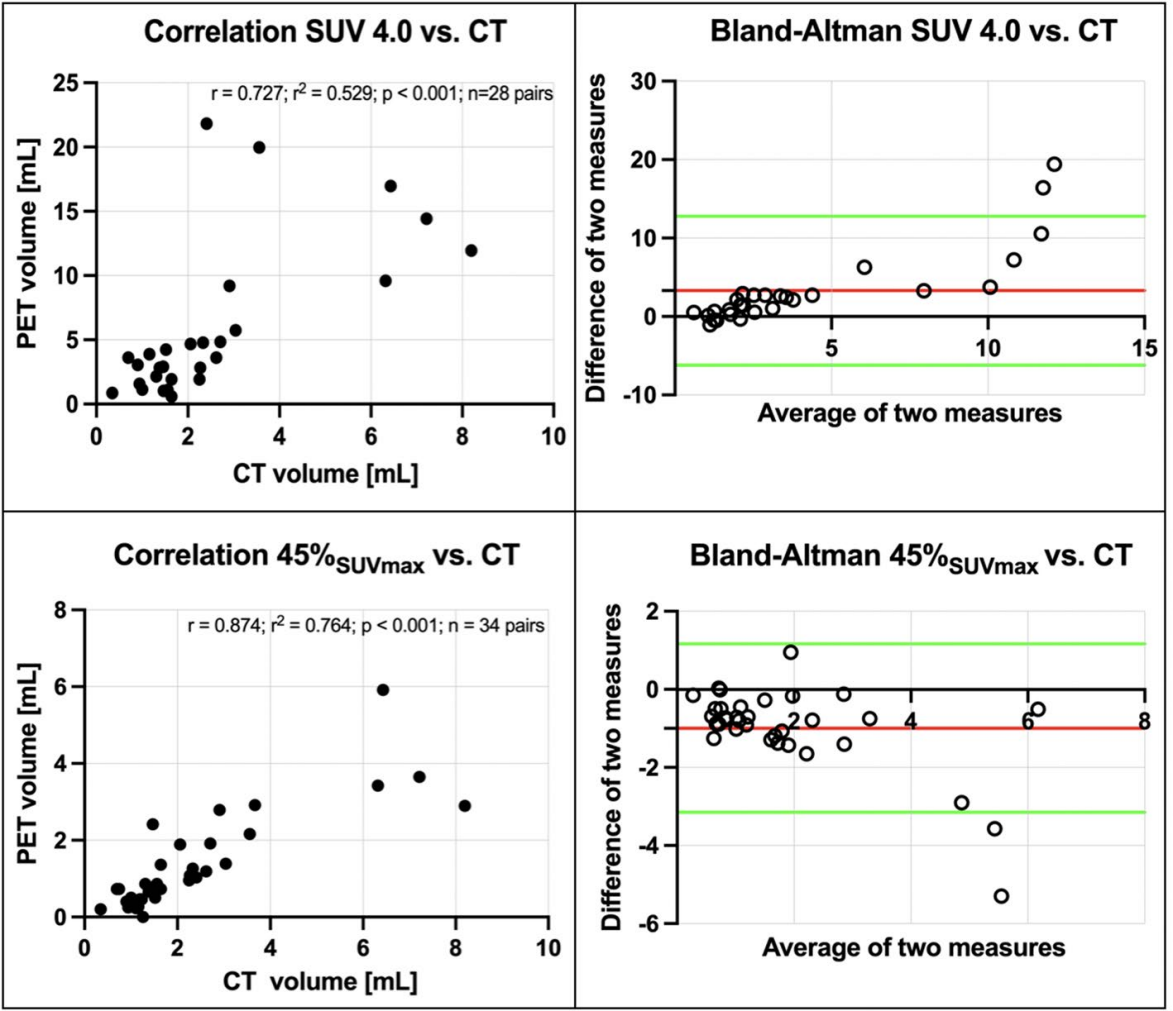
Correlation of PET volumes and CT-based reference standard. Upper row: PET volume SUV 4.0 (*r* = 0.727; r^2^ = 0.529; *p* < 0.001; *n* = 28 pairs). Lower row: PET volume isocontour of 45% SUV_max_ (*r* = 0.874; r^2^ = 0.764; *p* < 0.001); each correlation plot is accompanied by the corresponding Bland-Altman plot (red line: mean difference of two measures; green lines: mean difference of two measures ± 1.96 * standard deviation of the mean difference

**Fig. 2:**
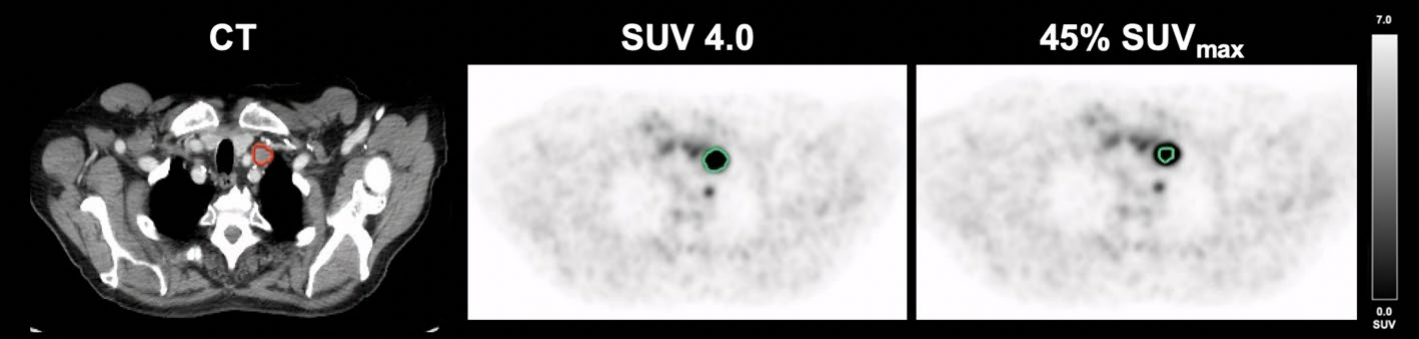
Delineation methods in a lymph node metastasis of a patient with GEPNET. Volumetric reference standard 2.91 mL; SUV 4.0: 9.19 mL; 45% SUV_max_: 2.79 mL

As demonstrated in Fig. 1, correlation and Bland-Altman plots are employed to illustrate the optimal correlation between PET- and CT-derived volumes. An illustrative case is presented in Fig. 2.

### Individual backwards thresholding

The backwards thresholding revealed a corresponding fixed SUV threshold of 8.9 ± 5.3 and isocontour relative to SUV_max_ of 32 ± 10%. The resulting CoV was 59.6% (fixed SUV threshold) and 31.3% (isocontour relative to SUV_max_) among all 34 lymph node metastases. However, results demonstrated a slightly lower correlation coefficient (SUV 8.9: *r* = 0.617; r^2^ = 0.380; *n* = 34 pairs; *p* < 0.001; 32.0%: *r* = 0.718; r^2^ = 0.515; *p* < 0.001; *n* = 32 pairs; Table 1) compared to the analyses above after applying these resulting mean values of backwards thresholding to all 34 lymph node metastases and correlating these volumes to the CT-derived volumetric reference.

## Discussion

In the context of tumor delineation, the most logical volumetric reference is that provided by liver metastases, given their high frequency in GEPNET. However, several factors limit the utility of this approach. These include the high physiological liver uptake observed in SSTR PET, the potential for diffuse infiltration, and difficulties in distinguishing lesion margins. By comparison, lymph node metastases offer a more clearly delineable, measurable disease, making them better suited for evaluation.

The findings of this pilot study suggest that an isocontour-based approach with 45% lesional SUV_max_ demonstrates the best correlation with CT-derived volumes, surpassing the efficacy of fixed SUV thresholds. This adaptive strategy is particularly beneficial in cases exhibiting variable SSTR expression across different lesions, thereby ensuring the inclusion of lower-expressing yet positive lesions. Based on the correlation curve, a potential outlier was identified. After performing the Grubbs outlier test, this value was confirmed to be a significant outlier. A subsequent correlation analysis, excluding this outlier, resulted in *r* = 0.864 (r^2^ = 0.746; *p* < 0.001), which is still higher than the correlations obtained with other tested values.

Conversely, the utilization of fixed SUV thresholds has the potential to exclude lesions exhibiting lower but biologically significant uptake, thereby resulting in an underestimation of the tumor burden. It is noteworthy that no SUV thresholding based on reference organs was employed in this study. The liver, a common reference region, poses certain challenges due to its heterogeneous metastasis load, especially in this cohort. Consequently, alternative approaches, such as the formula-based method proposed by Seifert et al. for [^68^Ga]Ga-PSMA PET, which relies on liver references, may also be subject to bias in cases of high hepatic tumor burden (Seifert et al. 2020).

This study was deliberately restricted to singular lymph node metastases, which allow for a robust definition of ground truth. In contrast, liver and bone metastases often appear less clearly delineated on CT and thus pose challenges for establishing a reliable morphological reference. Subsequent studies are planned to validate and extend the approach of this study to these additional metastatic sites.

The definition of a single optimal delineation method remains challenging, as PET is unable to fully resolve tumor heterogeneity at a cellular level. However, a method that closely approximates a reference standard enhances comparability and enables uniform, cross-institutional application. The absence of reliance on a reference organ also serves to minimize bias when SSTR expression changes due to systemic therapies (Cherk et al. 2018). The application of a backwards thresholding approach revealed considerable variability, as evidenced by the high coefficient of variation (59.6% for fixed SUV, 31.3% for isocontour relative to SUV_max_). Additionally, the 45% to SUV_max_ isocontour-based method demonstrated superior correlation compared all other methods tried. Furthermore, in the context of alternative fixed thresholds or isocontour-based thresholds, it was observed that not all *n* = 34 pairs were correlated, given the unavailability of adequate volumetric assessments due to the delineation of half the body, e.g. by using a fixed SUV of 3.0. The potential for lymph node metastases to be small, and the subsequent potential for PET resolution limitations and spill-over effects, particularly after therapy when lesions shrink further, is a key consideration (Cysouw et al. 2016). This could introduce uncertainty in small-volume measurements, though the overall impact on total tumor burden may be limited. Further studies are needed to assess these effects.

It is important to contextualize our findings regarding the optimal threshold for PET-based lesion delineation. The 42% threshold method was originally developed based on studies using small, homogeneous spheres in phantom models with no background activity, specifically to correct for spillover effects (Burger et al. 2014). It is therefore not surprising that our analysis, which focused on homogeneous, highly active, non-bulky lymph nodes, identified a threshold close to 45% as the most accurate. However, it is well known that such a threshold approach does not generalize to larger, bulky or partially necrotic tumors, as demonstrated in previous studies across different tracers and tumor types (Tatewaki et al. 2021; Tibdewal et al. 2021; Liberini et al. 2021). In these heterogeneous lesions, fixed percentage thresholding significantly underestimates or misrepresents the true tumor extent due to areas of necrosis, variable uptake and partial volume effects. Accurate delineation of lymph node metastases is particularly important for tumor volume assessments, since PET-derived volumes often extend beyond anatomical boundaries due to their functional nature. This study proposes a method to identify the optimal threshold for achieving congruence between PET- and CT-derived tumor volumes in non-bulky, homogeneously active nodes. Therefore, it is crucial to clearly state that our results are only valid for non-bulky, homogeneously active lymph nodes, which limits the generalizability and clinical applicability of the results.

Nevertheless, our study provides important value by systematically confirming the performance of a simple and easy-to-implement thresholding approach in a specific, clinically relevant subgroup, namely non-bulky, metabolically homogeneous lymph node metastases. This provides practical guidance for routine clinical workflows where rapid and standardized segmentation is often required, particularly in the setting of small-volume disease. Furthermore, by clearly delineating the limitations of fixed-threshold methods, our work highlights the need for adaptive or multimodal segmentation strategies in more complex lesion types, providing important insights for future method development and clinical application.

In order to validate the 45% lesional SUV_max_ threshold, further research is required in the form of larger studies evaluating TTV across a range of metastatic sites. However, it is important to note that longitudinal studies are necessary in order to confirm the threshold’s robustness for therapy monitoring. It is also important to note that the study focused only on SSTR-avid metastases, meaning that patients with SSTR-negative lesions may have had their TTV underestimated, thus necessitating FDG PET for comprehensive assessment (Tian et al. 2022; Zhang et al. 2018). The Bland-Altman plot shows increasing inaccuracy for larger lesions when applying the 45% lesional SUV_max_ threshold. In larger lesions, the PET signal intensity can reach a level that is so pronounced as to obscure tumor boundary.

This study did not include patients with NEC; therefore, FDG PET/CT, which is commonly used in NEC – was beyond the scope of our analysis and not available in this cohort. The results of this study should not be extrapolated to NEC.

The study has several limitations that should be considered when interpreting the results:

The number of included lesions is relatively small. This pilot study was designed to develop and test a delineation concept in a well-defined setting. Although a larger patient cohort and multicenter validation would clearly strengthen the evidence, this is currently difficult to achieve, as [^18^F]SiTATE is a novel, not yet approved tracer with limited availability and without standardized production guidelines. Furthermore, only a minority of NET patients present with singular, clearly demarcated lymph node metastases that fulfill the stringent inclusion criteria applied here. The absence of unified standards for [^18^F]SiTATE PET production and implementation hinders multicenter studies, thus, harmonization efforts are essential to enable broader comparisons. Further limitations that should be addressed include the impact of bulky metastases, the accuracy of delineating lesions adjacent to reference organs, and the challenges of liver metastasis segmentation. Although minor inaccuracies in high tumor burden settings may not significantly alter total TTV, these aspects need further investigation. Differences between PET scanners and reconstruction protocols may also introduce variability, emphasizing the need for standardized imaging protocols or EARL accreditation (Filss et al. 2017). Histological confirmation of lymph node metastases was not obtainable in all cases. Nevertheless, the diagnosis of NET or a high clinical suspicion was confirmed before or following the respective PET imaging. It is recommended that future studies investigate TTV in therapy monitoring and different metastatic sites to refine quantitative PET assessment in GEPNETs. Additional studies are needed to propose delineation methods for visceral metastases. Multiple time-point analyses are planned for future studies, with the aim of validating and substantiating the present findings.

Accurate tumor delineation in SSTR PET/CT is imperative for quantifying tumor burden, evaluating therapy response, and informing clinical decision-making in GEPNET patients.

This study represents a first step toward standardized tumor delineation in [^18^F]SiTATE PET, presenting a dedicated approach for the segmentation of lymph node metastases. Our data demonstrate that an isocontour-based approach using 45% of lesional SUV_max_ provides the strongest correlation with CT-derived lymph node volumes, surpassing the performance of fixed SUV thresholds. This method is adaptive to inter-lesional variability in SSTR expression and does not rely on reference organs, thereby minimizing potential bias from physiological uptake changes or systemic therapy effects.

## Conclusion

The findings of this study support the clinical applicability of an 45% isocontour-thresholding relative to lesional SUV_max_ for [^18^F]SiTATE PET-based tumor delineation; however, further multicenter validation is required to assess its reproducibility across different PET scanners and patient cohorts. In addition, longitudinal studies should investigate the role of TTV in therapy monitoring, particularly in patients with mixed SSTR-avid and SSTR-negative disease. Standardization efforts for [^18^F]SiTATE PET imaging are required to enable cross-institutional comparability and integration into clinical workflows.

## Data Availability

The datasets used and/or analyzed during the current study are available from the corresponding author on reasonable request.

## Abbreviations

GEP: Gastroenteropancreatic
NEC: Neuroendocrine carcinoma
NET: Neuroendocrine tumor
ROI: Region of interest
SAD: Short axis diameter
SSTR: Somatostatin receptor
SUV: Standardized uptake value
TTV: Total tumor volume
VOI: Volume of interest
